# Identification and characterization of intact glycopeptides in human urine

**DOI:** 10.1038/s41598-024-53299-3

**Published:** 2024-02-14

**Authors:** Fernando Garcia-Marques, Keely Fuller, Abel Bermudez, Nikhiya Shamsher, Hongjuan Zhao, James D. Brooks, Mark R. Flory, Sharon J. Pitteri

**Affiliations:** 1 Canary Center at Stanford for Cancer Early Detection, Department of Radiology, Stanford University School of Medicine, 3155 Porter Drive MC5483, Palo Alto, CA 94304 USA; 2 Department of Urology, Stanford University School of Medicine, Stanford, CA 94305 USA; 3 Cancer Early Detection Advanced Research (CEDAR) Center, Knight Cancer Institute, Oregon Health & Science University, Portland, OR 97239-3098 USA

**Keywords:** Glycobiology, Glycoproteins, Biomarkers

## Abstract

Glycoproteins in urine have the potential to provide a rich class of informative molecules for studying human health and disease. Despite this promise, the urine glycoproteome has been largely uncharacterized. Here, we present the analysis of glycoproteins in human urine using LC–MS/MS-based intact glycopeptide analysis, providing both the identification of protein glycosites and characterization of the glycan composition at specific glycosites. Gene enrichment analysis reveals differences in biological processes, cellular components, and molecular functions in the urine glycoproteome versus the urine proteome, as well as differences based on the major glycan class observed on proteins. Meta-heterogeneity of glycosylation is examined on proteins to determine the variation in glycosylation across multiple sites of a given protein with specific examples of individual sites differing from the glycosylation trends in the overall protein. Taken together, this dataset represents a potentially valuable resource as a baseline characterization of glycoproteins in human urine for future urine glycoproteomics studies.

## Introduction

Glycosylation of proteins encompasses an extraordinarily diverse post-translational modification class wherein complex carbohydrate structures are linked, principally via hydroxyl (O-linkage) and asparagine (N-linkage) attachments, to the side chains of polypeptide backbones. The glycan additions, in myriad branched-chain structures, are formed from building blocks of mannose, high mannose, fucose, and sialic acid and are assembled, modified, and removed by a highly regulated set of enzymatic activities in cells^1^. The diversity of glycosylation modifications on proteins contributes significantly to the estimated millions of proteoforms, or protein variant isoforms, estimated to comprise the full cellular proteome^2^. Since proteins are the direct effectors of most biological processes, a more thorough characterization of this modification class is critical to both a better understanding of cellular mechanisms and for discovery of new disease biomarkers.

At a functional level, emerging data indicates that protein glycosylation is integrated with fundamental biological processes including cell–cell recognition, signal transduction, and protein trafficking^3^, and protein glycosylation figures prominently in health and disease^1^. Multiple congenital disorders have long been associated with inborn glycosylation defects that often present with phenotypes including neurologic abnormalities and intellectual disabilities^4^. Furthermore, a growing body of evidence demonstrates critical linkages between protein glycosylation and cancer progression. For example, in serous ovarian carcinoma, glycoproteomic signatures can be used to classify disease subtypes that have distinct clinical outcomes^5^. Discrete classes of protein glycosylation have been observed in tissue specimens collected from patients with benign prostatic hyperplasia that can be distinguished from prostate cancer^6^. Separate studies in prostate cancer have identified specific glycoproteomic profiles that correlate with tumor aggressiveness and include markers potentially specific to metastatic disease^7,8^. The wealth of glycoproteomic data demonstrate that glycoproteins specifically mark cancers and suggest that these glycosylation changes may play critical roles in promoting important biological state changes that occur during tumor development, invasion, and metastasis, underscoring the importance of gaining a better understanding of their biological functions.

Glycoproteins, often secreted from cells and tissues and detectable in body fluids, also provide a rich source of noninvasive biomarkers for cancer detection. For example, non-invasive detection and treatment monitoring in pancreatic cancer diagnosis involves assaying for glycosylated proteins in the serum that are detected by the monoclonal antibody CA19-9 which is known to bind a specific sialyl glycan class^9^. Measurement of serum glycoprotein prostate serum antigen (PSA) is widely used to screen for and monitor prostate cancer. PSA demonstrates a number of glycosylation modifications, resulting in PSA “glycoforms”, that can differ between benign and malignant prostate tissues and have been proposed as candidate biomarkers that improve PSA performance as a screening biomarker^10,11^. While substantial biomarker discovery work has focused on plasma and serum from blood, much less effort has been directed at measuring biomarkers in more accessible fluid types such as urine. Urine has been shown to harbor a rich variety of modified proteins with over 2600 glycoproteins identified to date^12^. While urinary glycoproteomics is a logical frontier for discovery of biomarkers for urologic indications such as prostate, kidney and bladder cancers, glycoprotein alterations in urine have also been described for organs not in direct continuity with the urinary tract, including liver, lung and stomach cancers^12^.

Given the potential of urine glycoproteins as disease biomarkers, there is a need to develop improved urinary glycoproteomic workflows aimed toward biomarker discovery in this highly accessible biofluid. One roadblock of comprehensive glycoproteomic profiling in urine and other body fluids has been the inability of mass spectrometry (MS) and downstream analytic tools to effectively identify the vast diversity of attached glycan structures. To circumvent this challenge, MS-based glycoproteomic profiling efforts have often employed enzymatic removal of glycans prior to downstream MS, typically involving shotgun mode data acquisition on derivative tryptic peptide digests. In this approach, glycosylated amino acids retain a small chemical adduct after enzymatic deglycosylation that is detectable by MS. While these studies have provided a critical foundation by identifying glycosylated amino acid residues, including those in urine, the ability to understand more granular information encoded in the complex glycan structures themselves is forfeited because of the upfront deglycosylation step^13^. More recently, methods for characterization intact glycopeptides and determining structures for attached glycans using MS with companion bioinformatic tools like Byonic have enabled mass-based identification of a growing list of peptide-attached glycans^14^. In addition, the efficiency of glycoproteomic workflows continue to improve with the advent of chromatographic strategies including hydrophilic-lipophilic balance (HLB) and C18-faciliated reversed-phase chromatography modes and combined approaches^13,15, 16^, as well as hydrophilic interaction (HILIC) chromatography^17^, that have improved sample desalting and glycoprotein enrichment for urinary samples. Finally, choice of mass spectrometry configuration also has been shown to markedly impact the efficiency of glycopeptide detection. High-energy C trap-based dissociation (HCD) has emerged as an effective choice for peptide fragmentation improving the ability to detect and analyze structures of intact glycopeptides^18^. Here, we present an optimized workflow that combines elements of the sample processing steps and employs a new database of intact glycopeptides, resulting in an eightfold improvement in urinary glycopeptide detection over prior reports.

## Methods

### Glycoproteomics workflow

Pooled urine from healthy individuals was purchased from Innovative Research and 5 and 10 mL aliquots of pooled urine were each concentrated to 200 µL using 4 mL Amicon filters (Sigma-Aldrich) via multiple rounds of centrifugation (45 min, 3500×*g*, 4 °C). Filters were washed with an additional 3 mL of 50 mM ammonium bicarbonate (Sigma-Aldrich) and the concentrated solution was split into 3 aliquots of equal volumes. Each aliquot was adjusted to 120 µL with 50 mM ammonium bicarbonate solution followed by the addition of 12 µL or 14 µL of 10% sodium dodecyl sulfate (SDS, Invitrogen) for the initial 5 mL and 10 mL urine aliquots respectively. The disulfide bonds on cysteine residues on concentrated proteins were reduced with 5 µL of 200 mM Tris(2-carboxyethyl) phosphine (TCEP) (Sigma-Aldrich) at 70 °C for 1 h. The free thiol groups were alkylated with 7.5 µL of 200 mM iodoacetamide (Acros Organics) followed by an incubation of 45 min at room temperature in the dark. Proteins were precipitated with 1 mL of cold acetone and stored overnight at − 20 °C. Samples were centrifuged at 14,000×*g* for 10 min at 4 °C. Acetone was removed and the protein pellets were allowed to dry for 5 min. Pellets were reconstituted with 80 µL of 50 mM ammonium bicarbonate and vortexed. Urinary proteins were digested with 2 µg of sequencing grade modified trypsin enzyme (Thermo Fisher Scientific) for 18 h at 37 °C. The three separate 120 µL aliquots from each urine sample were combined, vortexed, and glycopeptides were enriched using strong anion exchange and electrostatic repulsion hydrophilic interaction chromatography (SAX-ERLIC) as described previously^19^. Briefly, the SOLA SAX solid phase extraction column was equilibrated with 3 mL of acetonitrile (Fisher Scientific), activated with 3 mL of 100 mM triethylammonium acetate (Fluka, Honeywell), and followed by adding 3 mL of 1% trifluoracetic acid (TFA, Sigma-Aldrich) in water (Fisher Scientific). 3 mL of equilibration solution consisting of 95% acetonitrile with 1% TFA in water were passed through the SOLA SAX column for equilibration. The combined tryptic peptides were diluted with 3 mL of equilibration solution, loaded, and passed through the column at a rate of 1 mL/min. Non-binding peptides were washed off with 6 mL of equilibration solution. Then, glycopeptides were eluted from the column by adding two 850 µL aliquots of 50% acetonitrile with 0.1% TFA in water followed by another two 850 µL aliquots of 5% acetonitrile with 0.1% TFA in water. The resulting glycopeptides were dried down using a speed vacuum (LabConco) and further fractionated using a high pH reversed-phase fractionation kit (Thermo Fisher Scientific) following manufacturer’s recommended protocol. The fractionated glycopeptides were dried down using a speed vacuum and reconstituted with 12 µL of 0.1% formic acid (Fisher Scientific) in HPLC MS grade water (Fisher Scientific) for LC/MS–MS analysis.

A Dionex Ultimate Rapid Separation Liquid Chromatography system (Thermo Fisher Scientific) was used to load 10 µL of the reconstituted glycopeptides onto a PEPMAP 100 C18 5 µm trap column (Thermo Fisher Scientific) with a flow rate set at 5 µL/min for 10 min. Glycopeptides were separated by reversed-phase chromatography on a 25 cm long C18 analytical column (New Objective) packed in-house with BEH C18, 130 Å, 1.7 µm particle size (Waters). An external column heater (MSWIL) was used to heat the analytical column to 60 °C. Glycopeptides were eluted by changing the mixture of mobile phase A (0.1% formic acid in water) and mobile phase B (0.1% formic acid in acetonitrile). The gradient program consisted of holding mobile phase B at 2% for the first 10 min, slowly ramped up to 35% over the next 85 min, followed by an increase to 85% over 5 min with a 5 min hold. The analytical column was re-equilibrated for 15 min prior to the next sample injection. The flow rate throughout the gradient was set to 0.3 µL/min. Eluted glycopeptides were analyzed using an Orbitrap Eclipse Tribrid mass spectrometer (Thermo Fisher Scientific). The cycle time was set at Top-speed for 3 s with an MS1 mass scan range of 375–2000 m/z and Orbitrap resolution of 120,000. The normalized AGC target was set to 250 percent and the maximum injection time to auto. The most abundant precursor ions were fragmented with higher energy collisional dissociation (HCD) and with a collision energy set to 38%. Dynamic exclusion was enabled for 15 s with the mass tolerance to 10 ppm. The normalized AGC target for the MS2 was set to 200%. MS2 fragments were detected in the Orbitrap with a mass resolution of 30,000 with injection time set to auto.

Byonic software 4.0.12 (Protein Metrics) was used to search raw files against a focused-human-urine protein database (2021; 2421) generated from shotgun proteomics of urine and the 309 mammalian N-glycan library provided in the Byonic software for glycopeptide identification. Parameters included trypsin digestion with a maximum of two missed cleavages and precursor mass tolerance of 10 ppm. Fixed cysteine carbamidomethylation and variable methionine oxidation, asparagine deamination, and N-glycan modification on asparagine contained within a N-X-S/T (where X can be any amino acid except proline) N-glycosylation amino acid consensus sequence were also specified.

### Data processing and analysis

Peptide identifications were filtered for Byonic Score greater than 150, and log probability greater than 1.5. The mass difference between two fucoses and one sialic acid is 1 Da and can lead to misidentifications of glycopeptides when the incorrect monoistopic peak is identified. We corrected for this problem by: (1) selecting glycopeptide identifications containing two or more fucoses where the mass accuracy was determined to be greater than − 1 Da, (2) determining the maximum number of sialic acids possible (= the number of hexoses minus 3), and (3) if the maximum number of sialic acids in the glycopeptide was less than the maximum number of sialic acids possible, two fucoses were replaced by one sialic acid in the glycopeptide identification.

Glycopeptides were classified according to the numbers of each combined sugar into seven glycan types (high mannose, hybrid, complex undecorated, complex sialylated, complex fucosylated, complex fucosylated plus sialylated, and other) using the decision tree in the Supplementary Fig. 1. All glycan structures containing two or less HexNAc, and three or less Hex were classified as “other”. If the number of HexNAc equaled two and the number of Hex was greater than three, the glycans were classified as “high mannose”. If the number of HexNAc was greater than or equal to 3, the number of Hex was greater than or equal to 3, and the number of Hex in the glycan main core was lower than HexNAc, the glycan was classified as “hybrid glycan”. For complex glycans: (1) those that did not contain Fuc or NeuAc, were classified as “complex undecorated”, (2) those containing NeuAc but no Fuc were classified as “complex sialylated”, (3) those containing Fuc but no NeuAc were classified as “complex fucosylated”, and (4) those containing both Fuc and NeuAc, were classified as “complex fucosylated plus sialylated”.

Each identified glycoprotein was quantified using each assigned identification and distributed according to each glycan type after correction (if needed), dividing each spectral count per protein and glycan type by the total number of spectral counts per glycoprotein.

To better understand the relationship between the total urine proteome and the urine glycoproteome, we compared our glycoproteome data and a reference urine proteome^20^ by applying an overrepresentation analysis using the protein annotations according to GO biological process, GO cellular component, GO molecular function, biological pathway, protein domain, and site of expression, using the total human proteome as background. The analysis included only protein categories with greater than four proteins, and adjusted p-value of enrichment lower than 0.01, in at least one the datasets. Using these same criteria, we analyzed the protein sets determined by significant correlation (*P* < 0.01) against each of the seven glycan types considered in the analysis.

## Results

### Characterization of urine glycoproteome

**Figure 1:**
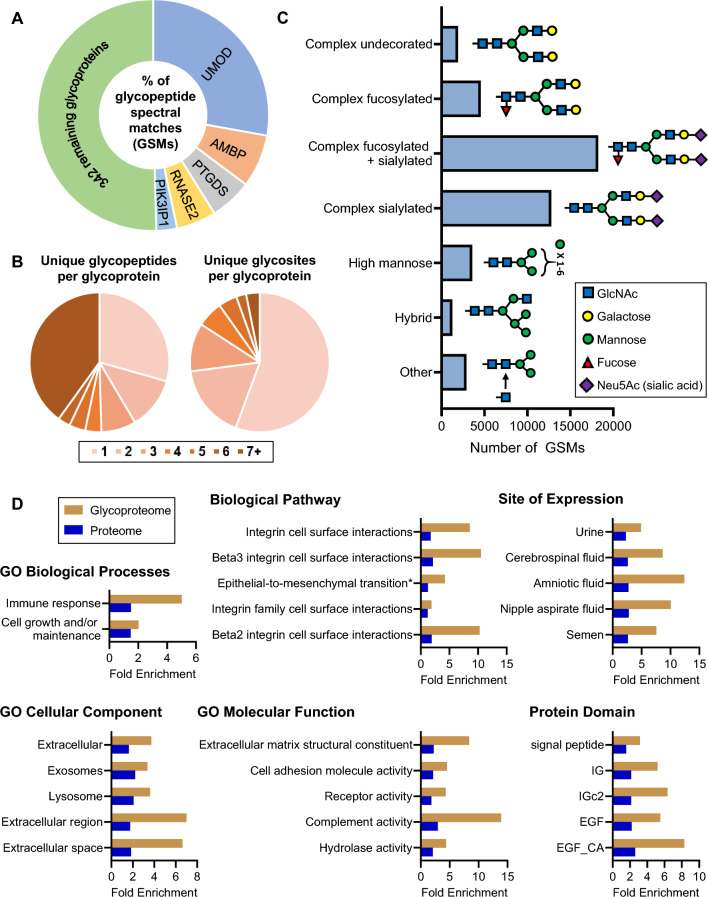
(**A**) Proportion of total identified glycopeptides (GSMs) corresponding to respective glycoproteins. (**B**) The number of unique glycopeptides identified per glycoprotein and the number of unique glycosites characterized per glycoprotein. (**C**) The number of glycopeptide spectral matches (GSMs) corresponding to different classes of glycans. An example putative glycan structure from each class is shown. (**D**) Gene enrichment analysis of identified urine glycoproteins compared to the urine proteome. All gene sets are significant (Bonferroni-corrected p < 0.01 in the urine glycoproteome and urine proteome unless otherwise noted). The top most significant gene sets in the urine glycoproteome are shown. *Not significant in the urine proteome.

Tandem mass spectrometry-based glycoproteomic analysis of pooled human urine samples collected from healthy individuals resulted in 45,303 total high quality intact N-linked glycopeptides (i.e. glycan attached to peptide backbone, GSMs). These glycopeptides corresponded to 8135 unique combinations of peptide sequences and glycan structures that mapped to 751 glycosites on 347 unique glycoproteins (Supplementary Table 1). Approximately 50% of the total identified glycopeptides corresponded to five abundant proteins (Fig. 1A). Uromodulin (UMOD), the most abundant protein in urine, accounted for ~ 28% of the total identified glycopeptides. Approximately 70% of the glycoproteins were identified by more than one unique glycopeptide (Fig. 1B). Furthermore, 44% of the glycoproteins had two or more unique glycosites for which we were able to characterize the glycan composition (Fig. 1B). The glycan compositions on the glycopeptides included more than 269 unique structures which were classified by glycan group (Fig. 1C, Supplementary Table 2). The complex decorated glycans (complex fucosylated, complex sialylated, and complex fucosylated and sialylated) were the most abundant glycan structures, with complex fucosylated and sialylated being the most abundant types. Complex undecorated, high mannose, hybrid, and other glycan structures were less abundant, comprising 21.3% of the total glycans.

To further study the biological properties of glycoproteins in urine, we performed gene enrichment analysis using Gene Ontology (GO) databases and biological pathway, protein domain, and site of expression databases. For each of these classes, enrichment analyses of the glycoproteins we identified and a large urine proteome dataset were performed^20^ (Fig. 1D, Supplementary Table 3). GO biological process including immune response and cell growth and/or maintenance were the most significantly enriched categories in the urine glycoproteome and showed higher fold enrichment compared to the urine proteome. Integrin-related cell surface interactions and epithelial-to-mesenchymal transition were the most highly enriched biological pathways in the urine glycoproteome. Not surprisingly in the urine glycoproteome, “urine” was the most significantly (lowest adjusted p-value) enriched site of expression, with cerebrospinal fluid, amniotic fluid, nipple aspirate fluid, and semen also showing significant enrichment.

The GO cellular components showed significant enrichment in urine glycoproteins for extracellular, exosomes, lysosome, and extracellular region/space, an expected finding since secreted proteins are commonly glycosylated. Molecular functions showed a significant enrichment in the urine glycoproteome dataset including proteins associated with the extracellular matrix structural constituents, cell adhesion molecule activity, receptor activity, complement activity, and hydrolase activity. Protein domains related to signal peptide, immune response (e.g. IG and IGc2) and EGF (a domain present on cell surface proteins) were found to be significantly enriched in the urine glycoproteome.

### Classification of glycoproteins by dominant glycan type

**Figure 2:**
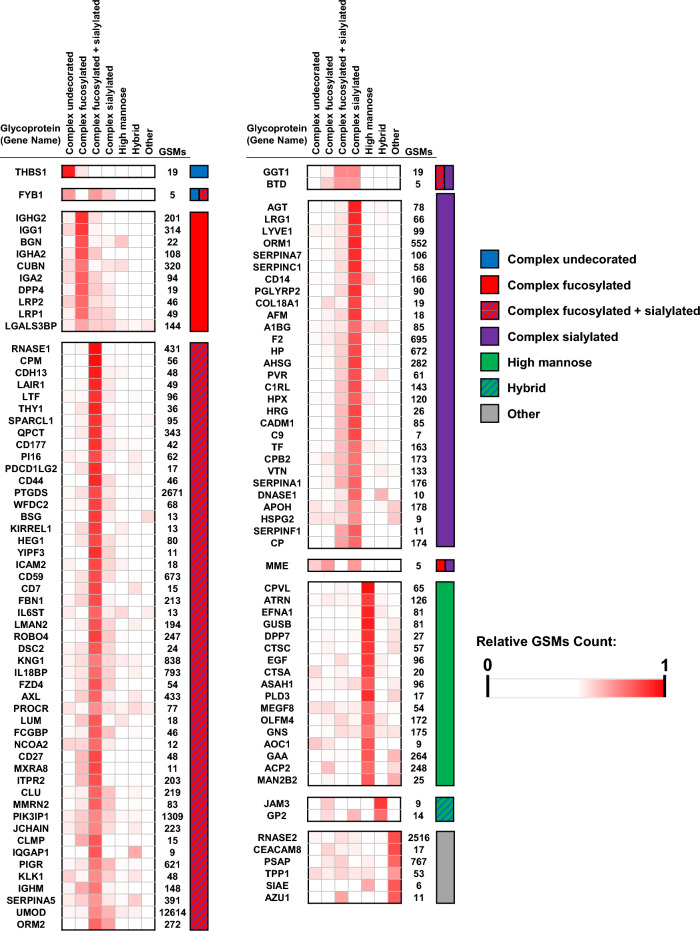
Heatmaps showing the distributions of glycan types identified on proteins. The relative intensity of each glycan class is calculated based on the number of GSMs for the corresponding glycopeptides. Proteins are clustered by dominant glycan class.

Glycoproteins can be classified by the predominant putative type of glycan (e.g. complex undecorated, complex fucosylated, complex sialylated, complex fucosylated + sialylated, high mannose, hybrid, or other). For each glycan type, the spectral counts were normalized to the total number of identified glycopeptides per protein and the glycoproteins were then clustered using a Pearson correlation analysis to display proteins with a significant correlation (*P* < 0.01) based on the glycan types (Fig. 2). For most of the glycoproteins (76%), complex structures were the predominant glycan type, and of the complex glycans, complex fucosylated and sialylated were most commonly observed (Figs. 1C and 2). Notably, four immunoglobulin proteins were found in the complex fucosylated glycoprotein cluster.

**Figure 3:**
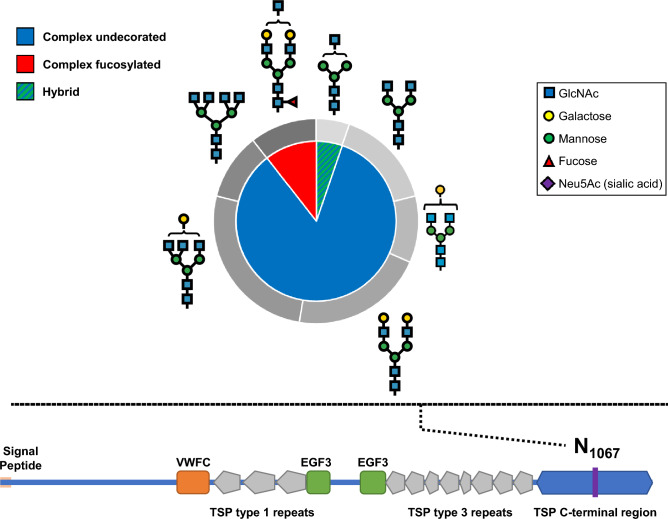
Glycans identified on asparagine- 1067 (N_1067_) of thrombospondin (THBS1). Classes of glycans are shown in the inner pie chart and the breakdown of glycans corresponding to the respective classes are shown in the outer doughnut chart. A schematic of the THBS1 protein sequence is shown at the bottom with the different protein domains and the purple line indicating the location of N_1067_.

The only protein with a complex undecorated dominant glycan type was thrombospondin-1 (*THBS1*), an adhesive glycoprotein that mediates cell-to-cell and cell-to-matrix interactions^21^. As shown in Fig. 3, we detected 19 total glycopeptides, 84% of which are complex undecorated glycans, that map to a single glycosite (ASN1067) in the C-terminal region of thrombospondin-1, with the remaining glycans including complex fucosylated (11%) and hybrid (5%) types. According to Interpro and Gene Ontology cross-reference databases, this glycoprotein domain is present in proteins involved in calcium binding and cell adhesion.

**Figure 4:**
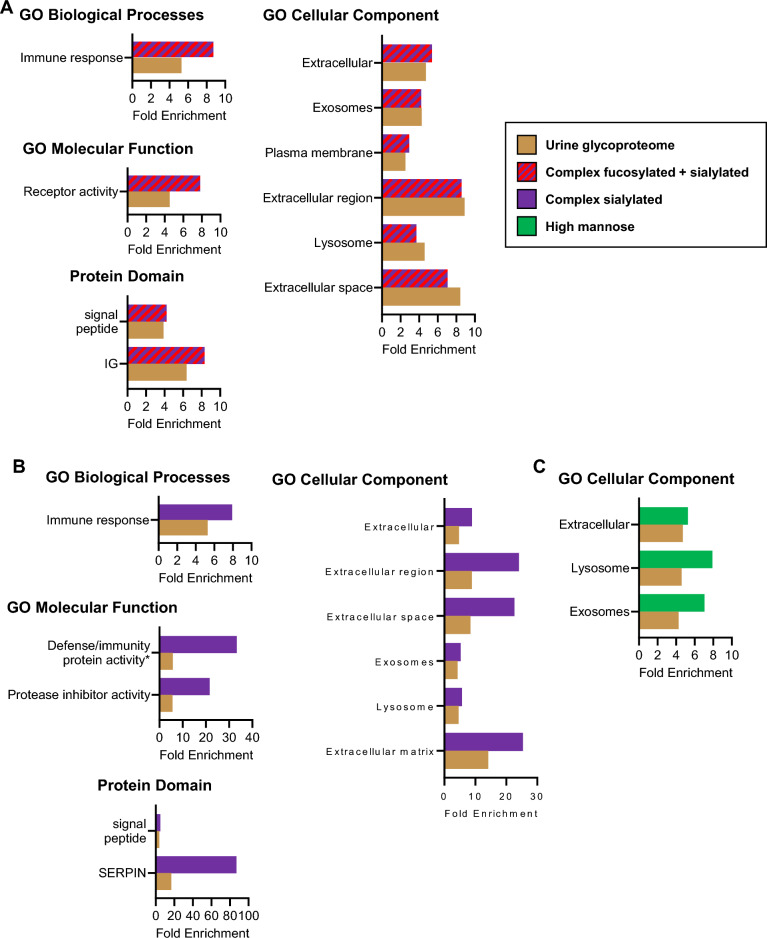
Gene enrichment analysis of glycoproteins with dominant (**A**) complex fucosylated and sialylated glycans, (**B**) complex sialylated glycans, and (**C**) high mannose glycans, compared to the overall urine glycoproteome. All gene sets are significant (Bonferroni-corrected p < 0.01 in the urine glycoproteome and urine sub-glycoproteome) unless otherwise noted. The top most significant gene sets in each urine subglycoproteome are shown. *Not significant in the urine glycoproteome.

Since the complex fucosylated and sialylated glycoproteins were the most common glycan type in the urine glycoproteome, we performed a gene enrichment analysis to compare these glycoproteins to the human proteome (Fig. 4A). We observed significant enrichment of proteins related to immune response. The complex fucosylated and sialylated glycoproteins were enriched for extracellular, exosomes, plasma membrane, and lysosome proteins, as well as molecular functions related to receptor activity.

Using the same approach, we compared complex sialylated glycoproteins, the second most abundant glycan type, to all human proteins (Fig. 4B). Once again, there was enrichment for glycoproteins involved in the immune response. Interestingly, complex sialylated proteins were enriched in similar cellular compartments compared to the complex fucosylated and sialylated glycoproteins, showing enrichment in extracellular, exosomes, and lysosome -related proteins. Complex sialylated proteins also showed significant enrichment in defense/immunity protein activity and protease inhibitor activity, as well as signal peptide and SERPIN (a specific type of protease inhibitor) domain.

When we compared predominantly high mannose glycoproteins to all human proteins (Fig. 4C), we did not observe significant enrichment in any GO biological processes, GO molecular functions, or protein domains. However, we did observe enrichment in subcellular locations of extracellular, exosomes, and lysosome.

### Identification and characterization of protein glycosites

**Figure 5:**
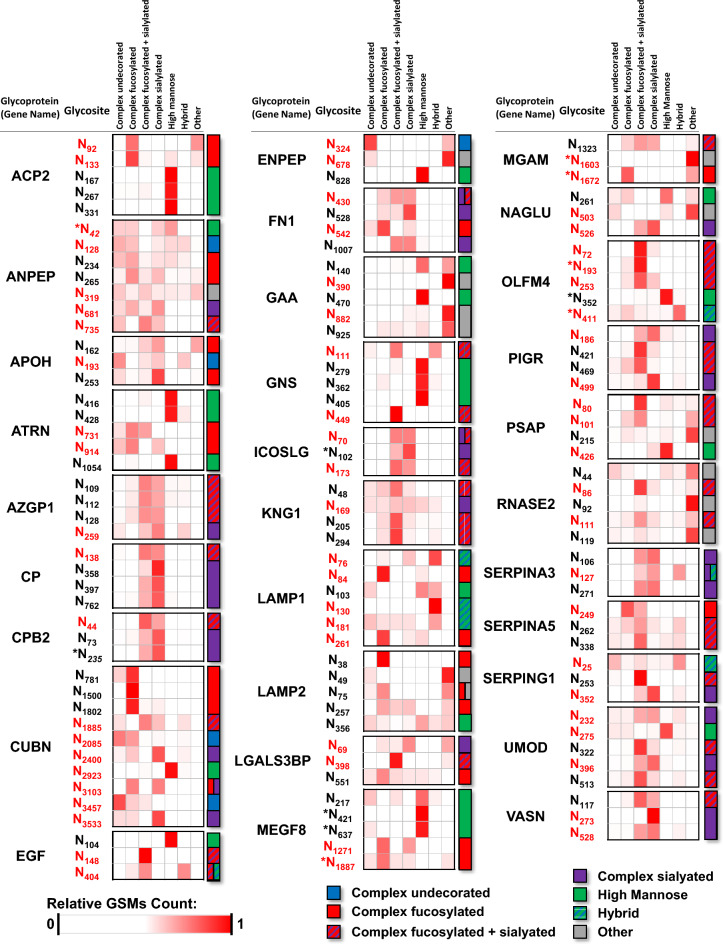
Heatmaps showing the distributions of glycan class by specific protein glycosite. The relative intensity of each glycan class is calculated based on the number of GSMs for the corresponding glycopeptide. Sites in red indicate that the dominant glycan class at the site is different than the dominant glycan class for the overall protein. *Indicates that the glycosite is not annotated in UniProt.

**Figure 6:**
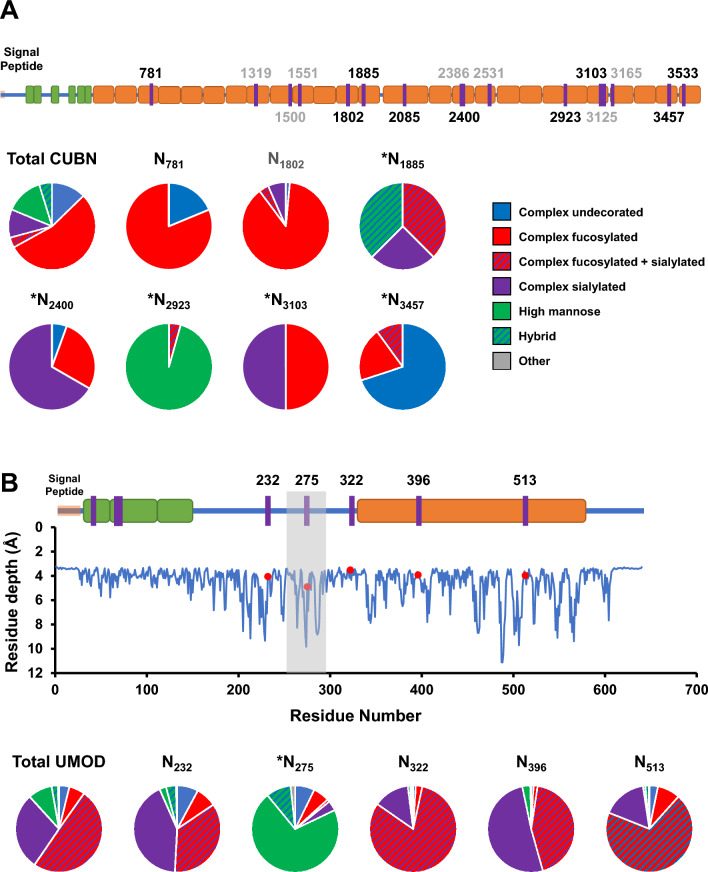
(**A**) Top: schematic of protein sequence for cubilin (CUBN). Glycosites are indicated by purple lines and numbers with gray numbers referring to glycosites that were not identified with more than two total glycopeptides. Green and orange indicate EGF-like and CUB domains respectively. Bottom: Pie charts indicate the composition of glycan types identified for the overall protein (Total) and at the individual sites. *Indicates that the dominant glycan type at the site differs from the overall dominant glycan type on the protein. (**B**) Top: schematic of protein sequence for uromodulin (UMOD). Red indicates ZP domain in UMOD. Middle: calculated residue depth for amino acids in UMOD. Red dots indicate characterized glycosites. Unless otherwise specified, information in (**A**) applies to (**B**).

The information provided by intact glycoproteomics analysis allows the identification of the specific amino acid that is glycosylated in a protein (i.e. glycosite) and characterization of the glycan composition at the glycosite. In this study, we evaluated the meta-heterogeneity^22^ (i.e. the variation in glycosylation across multiple sites of a given protein), to determine whether dominant glycan types differed between individual glycosites within a single protein. We compared the observed glycan species for the overall protein to the glycans observed at each individual glycosite. Figure 5 shows proteins (with three or more characterized glycosites) that have significant meta-heterogeneity, as defined by the dominant glycan type on one or more glycosite(s) differing from the glycosylation information from combining information across glycosites for a given protein. For example, cubilin (CUBN), an endocytic receptor important in metabolism by facilitating the uptake of lipoproteins, vitamins and iron^23–27^, shows predominantly complex fucosylated glycans when the protein is viewed as a whole (Fig. 6A). However, when examining the individual glycosites, CUBN showed a high degree of meta-heterogeneity with the dominant glycans for N_781_/N_1802_, N_2400_, N_2923_, and N_3457_ dominated by complex fucosylated, complex sialylated, high mannose, and complex undecorated glycans respectively.

An additional example of a protein exhibiting meta-heterogeneity in glycosylation is uromodulin (UMOD). UMOD is the most abundant glycoprotein in urine and contributes to colloid osmotic pressure, retards passage of positively charged electrolytes, prevents urinary tract infections, and inhibits formation of liquid containing supersaturated salts and subsequent formation of salt crystals^28^. We identified 12,614 total glycopeptides corresponding to 1485 unique glycopeptides, containing 269 unique glycan structures that mapped to five glycosites on UMOD (Fig. 6B). The overall protein was found to have the highest percentage of sialylated and complex fucosylated glycans, consistent with a recent study specifically characterizing glycans from UMOD in urine^29^ and which was consistent with four of the five glycosites. However, N_275_ exhibited a strikingly different pattern with 71% of all glycopeptide identifications contain high mannose structures. Interestingly, glycan structures at this site differed from a previous study^30^ which identified only high mannose structures at N_275_. To determine if the difference in glycosylation on this specific site may be due to amino acid’s location on the three dimensional structure of the protein, we calculated the residue depth^31^ of each glycosite as shown in Fig. 6B. Interestingly, N_275_ had a larger residue depth than the other four glycosites which were mapped to more solvent-accessible areas on the protein. These results are consistent with the previous observation that protein structure dictates formation of N-glycan type^32^. Therefore, it is possible that the dominant high mannose glycans on N_275_ may be, at least partially explained by the limited accessibility of the glycosite therefore restricting access to the glycans at that site by glycosotransferases.

Alterations in glycosylation of proteins such as cubilin and uromodulin can have profound implications for protein structural conformation, molecular interactions, and biological functions due to the pivotal role of glycosylation in protein folding, stability, trafficking, and receptor-ligand interactions^3^. In the case of cubilin, a protein expressed in renal and intestinal cells, and a receptor involved in renal reabsorption and cellular transport, changes in glycosylation patterns can impact its ligand-binding affinity and transport efficiency^33^. Modulations in the glycosylation profile of cubilin may disrupt the proper recognition and binding of specific ligands, potentially compromising its role in renal transport processes and overall renal function^33^. Similarly, uromodulin, is a glycoprotein exclusively produced in the kidney, where it participates in urine concentration regulation and kidney defense mechanisms^34^. Alterations in uromodulin glycosylation can affect its structural stability, intracellular trafficking, and interactions with other urinary components and proteins. These modifications may influence uromodulin's polymerization, ion transport regulation, and involvement in immune responses within the kidney.

The specific consequences of glycosylation changes in cubilin and uromodulin are likely dependent on the nature and site of the altered glycans. These changes can result in functional modifications, modulated protein–protein interactions, modified receptor binding affinities, and potential effects on intracellular signaling pathways. Elucidating the precise effects of these glycosylation alterations is crucial for comprehending the underlying mechanisms and their implications for physiological processes and diseases.

In summary, urine is a highly attractive sample type for developing clinical assays, and this study describes deep analysis of the urine glycoproteome spanning more than four orders of magnitude of dynamic range of protein abundance. We describe the protein composition of the urine glycoproteome and how that differs from the overall urine proteome. This study also provides detailed characterization of glycans on specific glycosites of identified proteins and examples of meta-heterogeneity in glycosylation are shown with possible explanation by residue depth. This dataset may also find utility as a resource for future studies as a baseline characterization of pooled normal human urine.

### Supplementary Information

Supplementary Figure 1.

Supplementary Table 1.

Supplementary Table 2.

Supplementary Table 3.

Supplementary Table 4.

Supplementary Legends.

## Data Availability

The mass spectrometry proteomics data have been deposited to the ProteomeXchange Consortium via the PRIDE partner repository with the dataset identifier PXD038923.
