# Supplementary figures and images for: Identification and characterization of intact glycopeptides in human urine
Source: Sci Rep. 2024 Feb 14;14:3716. doi: 10.1038/s41598-024-53299-3 (PMC10866872; doi:10.1038/s41598-024-53299-3)

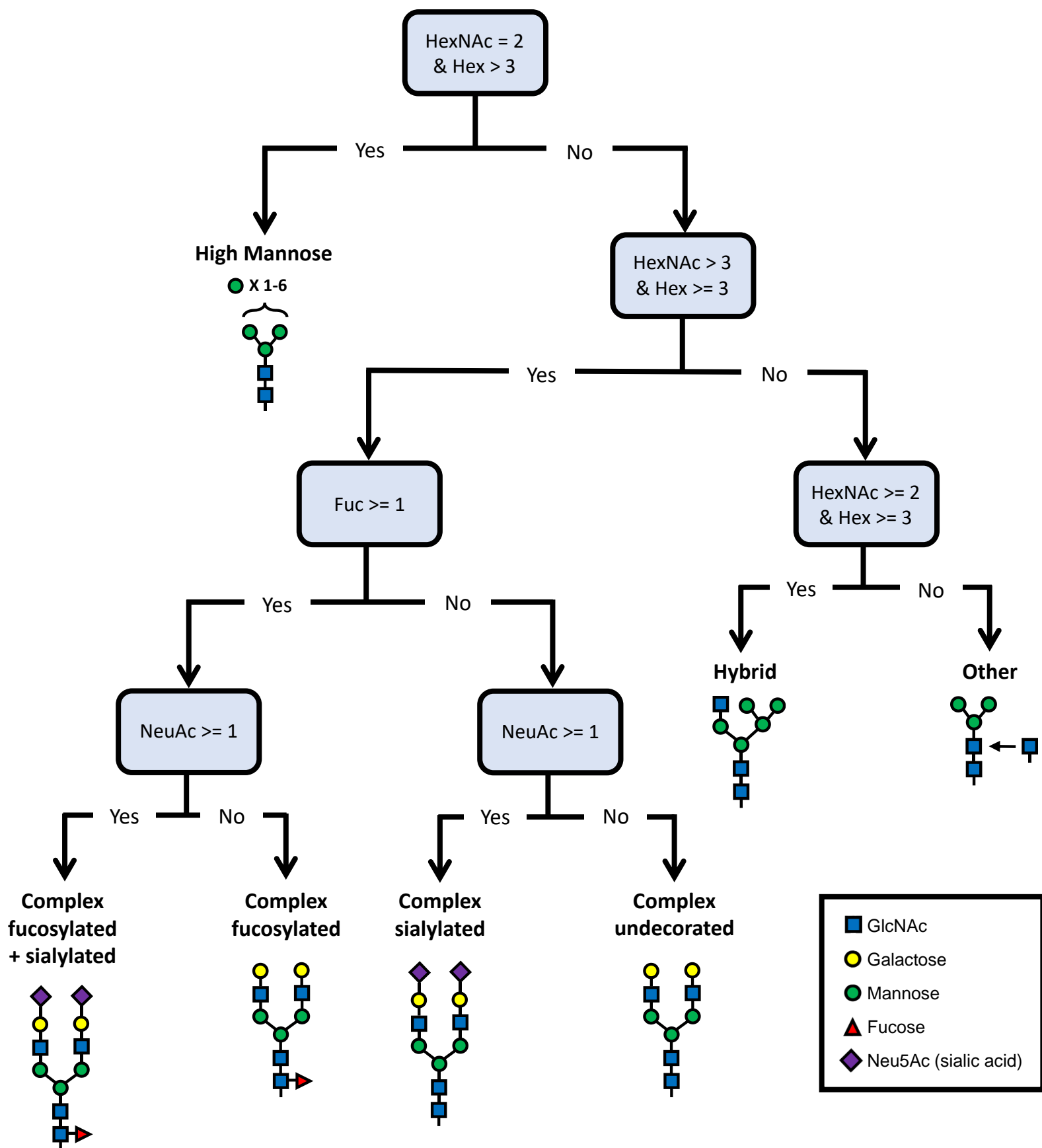

Supplement: Supplementary file 1 — Supplementary Figure 1. [file 41598_2024_53299_MOESM1_ESM.pdf]
