# Supplementary material for: Identification and characterization of intact glycopeptides in human urine
Source: Sci Rep. 2024 Feb 14;14:3716. doi: 10.1038/s41598-024-53299-3 (PMC10866872; doi:10.1038/s41598-024-53299-3)
Supplement: Supplementary file 6 — Supplementary Legends. [file 41598_2024_53299_MOESM6_ESM.docx]

**Supplementary Information**

**Supplementary Figure 1.** Decision tree for classifying glycans.
